# Integration of genomics and transcriptomics highlights the crucial role of chromosome 5 open reading frame 34 in various human malignancies

**DOI:** 10.18632/aging.205310

**Published:** 2023-12-07

**Authors:** Yilin Li, Yong Zhang, Dan Sun, Xiaofeng Zhang, Shangqin Long, Jiuxiang Feng, Zhongmin Wang

**Affiliations:** 1 Department of Gynecology, Dalian Women and Children’s Medical Center (Group), Dalian, China; 2 School of Clinical Medicine, Fujian Medical University, Department of General Surgery, Ningde Municipal Hospital, Ningde, China; 3 School of Clinical Medicine, Fujian Medical University, Department of Obstetrics and Gynecology, Ningde Municipal Hospital, Ningde, China; 4 Department of Obstetrics and Gynecology, Dalian No.3 People’s Hospital, Dalian, China

**Keywords:** C5orf34, biomarker, pan-cancer, genomics, transcriptomics

## Abstract

Although some data suggest that chromosome 5 open reading frame 34 (C5orf34) plays a pivotal part in the onset and disease progression of various cancers, there is no pan-cancer investigation of C5orf34 at present. This study sought to establish the predictive importance of C5orf34 in a variety of human malignancies and to understand its fundamental immunological function. In our research, we applied a combination of several bioinformatics techniques and basic experiments to investigate the differential expression of C5orf34, and its relationship with prognosis, methylation, single nucleotide variant, clinical characteristics, microsatellite instability, tumor mutational burden, copy number variation, and immune cell infiltration of several cancers from the database that is publicly available with the aim of identifying the potential prognostic markers. In this study we found that C5orf34 expression differed significantly among cancers types, according to the findings. The expression level of C5orf34 is markedly increased in the majority of malignancies when compared to normal tissues, which is significantly correlated with an unfavorable prognosis of patients. Immunohistochemical staining confirmed the findings that C5orf34 expression was remarkably up-regulated in a variety of gynecologic cancers. Moreover, C5orf34 expression was shown to be correlated with the clinical features of patients. C5orf34 was also found to be expressed with genes that code for the major immune suppressors, chemokines, immune activators, chemokine receptors, and histocompatibility complex. Finally, our study shows that C5orf34 has the potential to be employed as a prognostic biomarker. Moreover, it might regulate the immune microenvironment in a variety of malignancies.

## INTRODUCTION

According to World Health Organization (WHO) estimations in 2019, cancer is the primary or second leading cause of death in many nations for people with an age less than 70. According to the most recent epidemiological investigation, new cancer cases that were diagnosed in 2020 were estimated to be 19.3 million, with the worldwide cancer burden anticipated to reach 28.4 million cases in the year 2040. Hence, this will be an increase of 47% from 2020 [1]. Furthermore, according to statistics, cancer inevitably leads to a high death rate, killing about 10.0 million people globally in the year 2020. As a consequence, the cancer incidence burden together with the mortality rate is constantly increasing globally [1]. In a nutshell, cancer is the leading cause of mortality and a major barrier to increasing life expectancy in every nation. In 1995, a publication on gene expression analysis of tumors gave a fresh viewpoint on cancer research [2]. There is a consensus that cancer is one of the obstacles that mankind has attempted to tackle. Currently, scientific developments have resulted in the creation of numerous gene expression signatures profiles, which have improved the diagnosis and prognosis of various sickness phases as well as the discovery of new therapeutic targets specific to certain disease states. Today, several studies have discovered that gene expression is correlated with tumor growth and prognosis today [3]. A growing number of studies on gene expression in prognosis and cancer progression have been conducted, and the research is still ongoing.

C5orf34 (chromosome 5 open reading frame 34) is a human gene that encodes the C5orf34 protein, which was among the genes with significantly altered expression [4]. C5orf34 protein is highly conserved across species with no tissue specificity and is expressed in several tissues, including the testis. It may be correlated with kinase-related cellular processes, according to structure-based studies. Furthermore, as cell proliferation and gene regulation are two key signal transduction trails that include nuclear kinase proteins, C5orf34 has been hypothesized to be nuclear [5]. As a result, it may play a crucial role in gene regulation and cell proliferation [5]. C5orf34 gene has the potential to serve as a clinical biomarker, according to Pensee Wu et al. [6], who detected differential methylation in eight of eleven women who developed gestational diabetes compared to matched controls. Other research has discovered that the rs209443540 mutation in C5orf34 can improve a cow’s capacity of sustaining milk production [7]. This mutation is defined by a replacement of G in the protein sequence resulting in a transition from glycine to serine [7]. C5orf34 may play a considerable part in facilitating the onset and progression of lung adenocarcinoma (LUAD) via the mechanism of modulating the MAPK signaling pathway [4]. However, the significance of C5orf34 in other tumors has not been identified; the relationship between C5orf34 and diverse malignancies warrants more research.

With the aid of the R software, we intensively explored the association of patients’ prognoses with the expression level of C5orf34 as well as its possible function in tumor immunity. Our data came from a variety of sources, including fundamental experiments, web tools, and public databases.

## MATERIALS AND METHODS

### C5orf34 expression analysis

ONCOMINE (http://www.oncomine.org/) is a robust genome-wide expression analysis service provided by a translational bioinformatics service [8]. The information was gathered to probe into how the expression level of C5orf34 differed among cancer types. The significance levels in this investigation were established at a *p*-value of 0.05, a gene rank in the top 10%, and a fold change of 2.

The Cancer Genome Atlas (TCGA) was used for the purpose of collecting profile data of expression from related cancers and adjoining normal samples, together with information outlining related clinicopathological features, from a database that is of landmark cancer genomic comprising huge cancer samples information covering 33 cancer types [9]. The abbreviations of the TGCA pan-cancer names were shown in Supplementary Table 1. GTEx [10], a large public database, was used to enrich tissue-specific normal samples in addition to those obtained from TCGA. For the purpose of performing pan-cancer analysis, we used software to extract and combine C5orf34 expression levels. The “wilcox.test” algorithm in R was used to investigate the differences in mRNA expression between cancer types in depth [11]. We also integrated TCGA database and GTEx database to further study the differences between normal tissues and cancer tissues using the R-package “ggpubr”.

### Immunohistochemical (IHC) analysis

At the Ningde Municipal Hospital of Ningde Normal University in China, we collected paracancerous tissues and tumor tissues from CESC, UCS, UCEC, and OV, and 10 tumor type pairs in each were collected to validate the difference of expression of C5orf34. The Human Ethics Committee at Ningde Municipal Hospital of Ningde Normal University, China, granted the experimental procedure, and the approval reference number is 20220203. After deparaffinization, epitope retrieval, and hydration, 3% hydrogen peroxide was used to inhibit endogenous peroxidase activity in the slices for 15 minutes. The slides were then placed in a humidity box and treated overnight at 4°C with the primary antibody C5orf34 (1:400, Novus, USA), followed by placing in a secondary antibody. Finally, diaminobenzidine was used to view the slides, which were then counterstained with hematoxylin. An Olympus BX63 microscope was utilized to examine immunohistochemical sections, and Image J software was employed for quantitative analysis of slides. The mean and standard deviation were adopted to represent the data. The SPSS 21.0 statistical program was used to conduct the statistical analysis.

### C5orf34 expression correlation to clinical phenotype and prognosis

Similar to previous study [12], we conducted an analysis using survival data sourced from the TCGA database to explore the relationship between clinical outcomes and the expression of C5orf34. The association between mRNA expression levels and patient survival rates was evaluated using disease-free interval (DFI), disease-specific survival (DSS), overall survival (OS), and progression-free interval (PFI). Kaplan-Meier and Cox analyses were performed for the purpose of investigating the correlation between C5orf34 expression and survival outcomes in patients with various cancer types. The R software (version: 4.0.3) was used to plot KM curves and forest plots. Following that, we performed a correlation study of clinicopathological parameters such as tumor grade, tumor stage, gender, age, race, and tumor status with the aid of R’s “limma” and “ggpubr” packages. The R software package “survminer” and “survival” were used to create the survival curves. The significance level was set at *p* < 0.05.

### The C5orf34 methylation and prognosis correlation

DNA methylation is widely established to play a significant role in a variety of malignancies. The relationship between patient prognosis and C5orf34 methylation in different malignancies was investigated in this study. The methylation data were acquired in its raw form from the TCGA database. The log-rank test and Kaplan-Meier tests were conducted to examine the relationship between survival outcome and C5orf34 methylation in patients with various cancer types.

### C5orf34 mutation analysis in a variety of cancers

In clinical practice, gene mutations, including gene single-nucleotide variant (SNV) and copy number variation (CNV), are common in almost all cancers. Thus, we performed C5orf34 mutation analysis in an open platform that visualizes and analyses gene copy number variations and mutations in a variety of cancers known as CBio Cancer Genome Portal [13]. Then, with the aid of the R packages “survminer” and “survival,” the correlation between patient prognosis and CNV was explored by log-rank tests and Kaplan-Meier analysis. The SNV is a single nucleotide at a specific chromosomal location. It is the most prevalent sort of genetic variation, and it has been proven in a variety of genomic locations for a long time [14]. Another study on the correlation between prognosis and SNV was conducted using a similar manner.

### Association of immunity with C5orf34 expression in pan-cancers

To begin, Sangerbox website [15], a useful online platform for TCGA data analysis, was used to investigate the relationship between immune-related genes and C5orf34 expressions, such as immune inhibitors, chemokine ligands, chemokine receptors, immunostimulators, and genes encoding major histocompatibility complex genes. The “limma” package in R was used to perform the co-expression analysis of C5orf34 and immune-related genes, and the results were visualized using the “RColorBrewer” and “reshape2” packages in R software. Following that, the CIBERSORT-ABS, QUANTISEQ, TIMER, EPIC, MCPCOUNTER, CIBERSORT, and XCELL algorithms were used to determine the relative proportion of certain immune cells [16]. The close association of immune cells infiltration with C5orf34 expression was then investigated thoroughly.

### Tumor mutation burden (TMB), microsatellite instability (MSI), and C5orf34 gene expression correlation analysis

To further investigate the immune-predictor significance of C5orf34 in pan-cancer, we investigated two new immunotherapy parameters: MSI and TMB. We hypothesized that C5orf34 expression in relation to TMB and MSI would indicate that a greater degree of genomic instability, as described by the MSI and TMB, may result in heightened immune-surveillance possibilities.

### Pan-cancer stemness indices and tumor microenvironment

Tumor cells, extracellular matrix, and mesenchymal cells make up the majority of the tumor microenvironment. These play a crucial role in the onset, progression, invasion, and metastasis of tumors [17]. The correlation between the proportion of stromal cells and immune cells and C5orf34 expression in TCGA tumor samples cells was investigated by means of the ESTIMATE method. The ESTIMATE score is based on gene expression features, which can indicate tumor purity; it also shows high accuracy in the estimate [18]. An analysis of the Spearman correlation was conducted between the C5orf34 expression level and the matrix score with the aid of the estimate and limma packages.

Melta used an approach known as the one-class logistic regression (OCLR) machine learning to identify epigenetic and transcriptomic feature sets from non-transformed pluripotent stem cells together with their differentiated posterity in a paper published in the journal “Cell” in 2018 [19]. The RNA stemness scores (RNAss) and DNA stemness scores (DNAss) were calculated based on epigenetic signatures and OCLR-based transcriptomic markers on all pan-cancer 33 TCGA cohorts, which were also highly correlated with immune checkpoint activation, immunocyte infiltration, and tumor heterogeneity. As a result, we investigated the correlation between DNAss/RNAss and C5orf34 expression in a comprehensive manner.

### Prediction of targeted miRNAs of C5orf34 and single-cell analysis of C5orf34 in female reproductive system tumors

The miRNet [20], miRTarBase [21], and StarBase [22] databases were employed to predict the miRNAs closely related to C5orf34. TISCH2 [23], short for the Tumor Immune Single Cell Hub 2, is a valuable repository of single-cell RNA sequencing data obtained from both human and mouse tumors. This resource facilitates a thorough analysis of gene expression within the tumor microenvironment (TME) across a wide spectrum of cancer types. GSE118828 [24] provided single-cell data of OV, GSE139555 [25] provided single-cell data of UCEC, and GSE168652 provided single-cell data of CESC. In the TISCH2 platform, we conducted single-cell type analysis for OV, UCEC, and CESC. More importantly, we analyzed the single-cell distribution characteristics of C5orf34 in the aforementioned female reproductive system tumors.

## RESULTS

### C5orf34 expression in human cancers at differential levels

**Figure 1:**
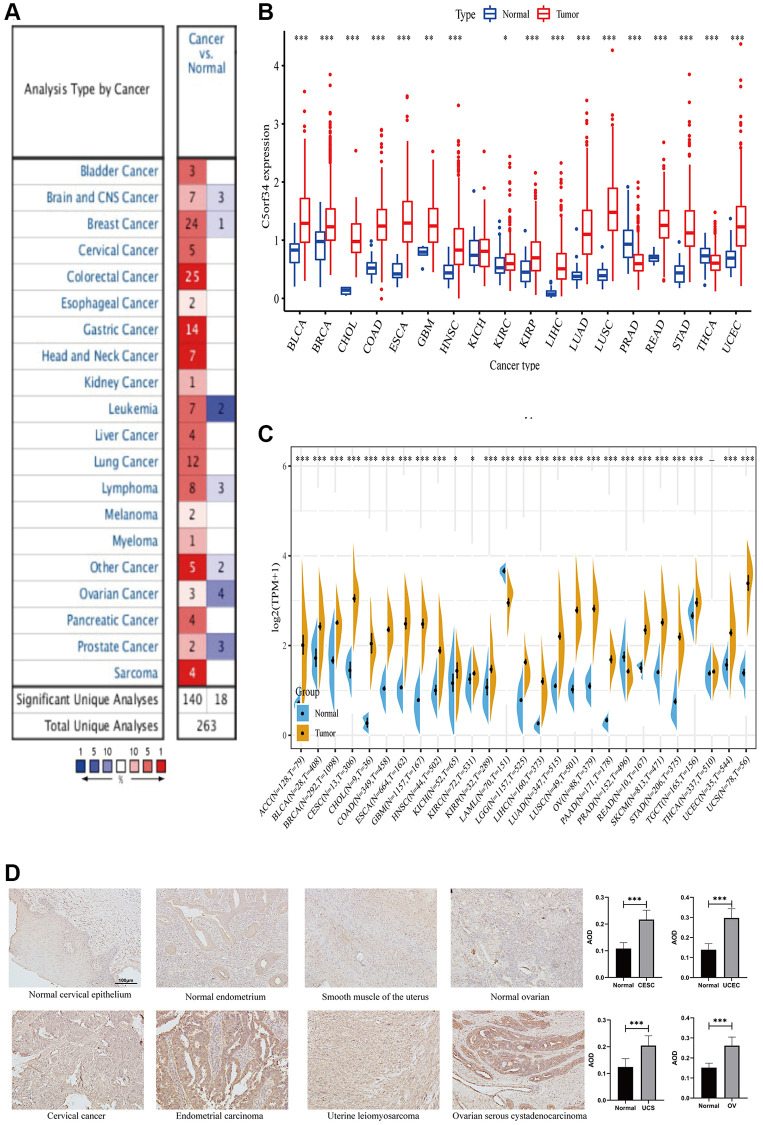
Comparison of C5orf34 expression level in several malignancies and its matching normal tissues based on (**A**) Oncomine platform, (**B**) TCGA cohort, (**C**) TCGA and GTEx cohorts. (**D**) Representative images and quantification analysis of C5orf34 immunohistochemistry in gynecologic cancers (^***^P < 0.001, ^**^P < 0.01, ^*^P < 0.05).

We used the Oncomine database for the purpose of examining C5orf34 expression in cancer tissues and normal tissues and discovered that its expression was notably higher in cancers than in healthy tissues (Figure 1A). C5orf34, on the other hand, was shown to be less expressed in several malignancies, including leukemia, prostate cancer, lymphoma, breast cancer, ovarian cancer, and central nervous system cancer (Figure 1A).

We then utilized the TCGA database to validate our findings. Similarly, C5orf34 mRNA level was considerably elevated in most malignancies such as CHOL, LIHC, ESCA, BRCA, READ, COAD, LUSC, STAD, LUAD, HNSC, BLCA, KIRC, KIRP, GBM, and UCEC in contrast with matched adjacent normal tissues (Figure 1B). In addition to THCA and PRAD, C5orf34 expression was found to be significantly elevated in the majority of cancers.

Due to the limited amount of paracancerous samples in the TCGA database, we combined data from TCGA and GTEx platforms to investigate the differential expression of C5orf34 in normal and cancerous tissues. In most cancers, the count of C5orf34 in tumor tissues was much greater than in normal tissues, which was in line with our earlier findings. C5orf34 expression level was also considerably lower in PRAD than in normal prostate tissues; however, there was no major difference between the expression of C5orf34 in normal thyroid tissues and THCA. Furthermore, in the case when normal tissue samples were added to LAML populations, the expression level of C5orf34 was lowered (Figure 1C). IHC results confirmed that OV, UCEC, CESC, and UCS tissues had higher C5orf34 expression levels than normal tissues, as shown in Figure 1D.

### C5orf34’s multifaceted prognostic value in human cancers

**Figure 2:**
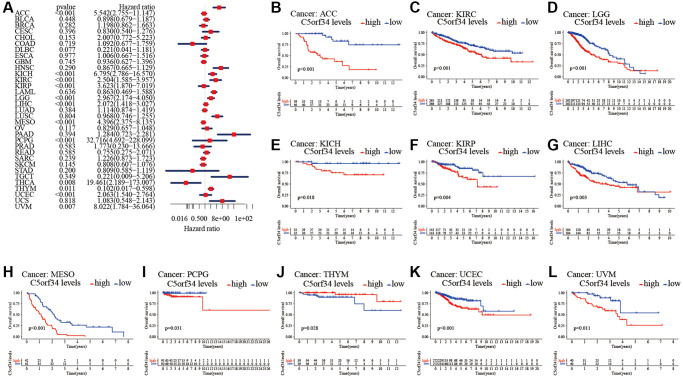
**Relationship of C5orf34 expression with the overall survival in pan-cancer.** (**A**) The forest plots of C5orf34 in pan-cancer’s overall survival; (**B**–**L**) C5orf34’s survival curves as regards overall survival of pan-cancer samples.

**Figure 3:**
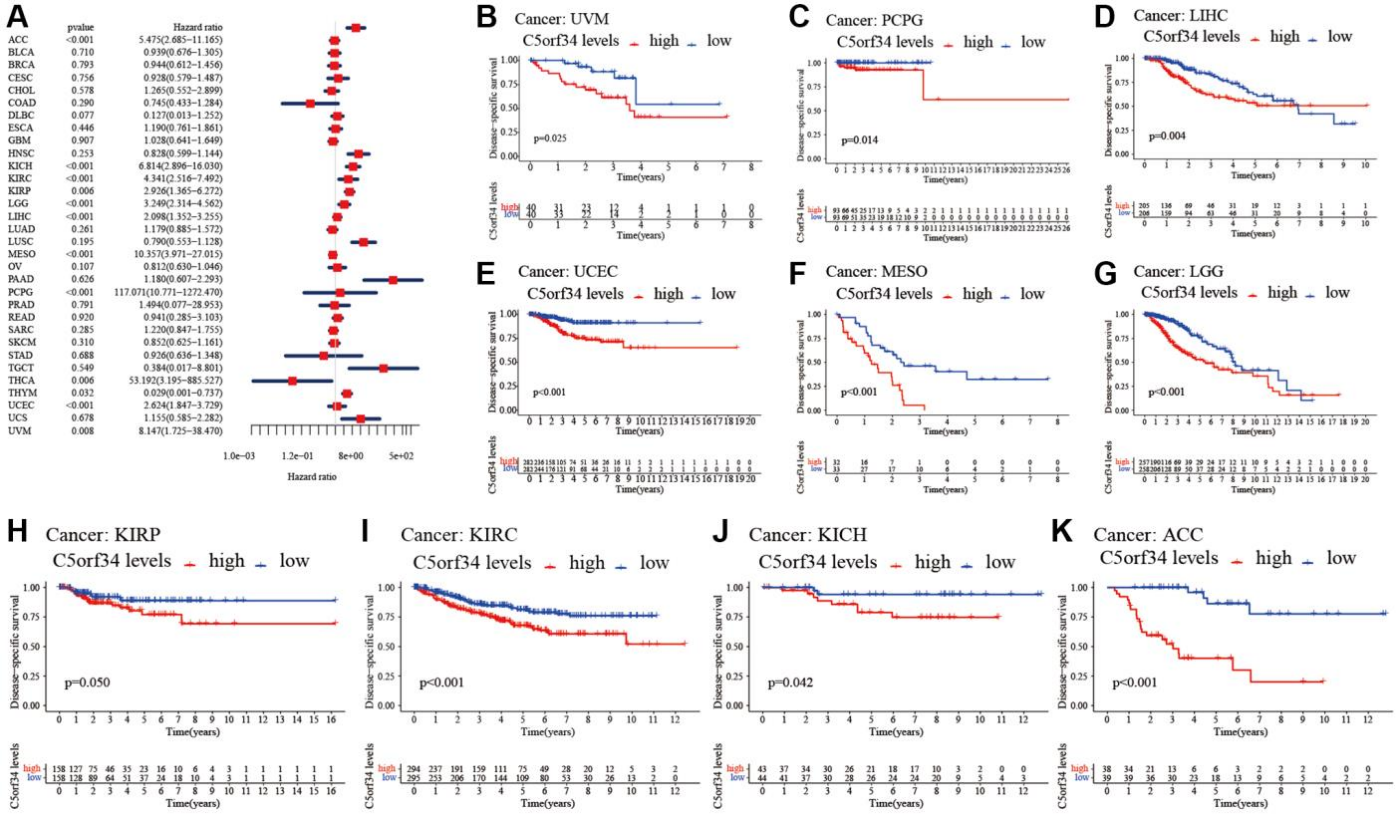
**Relationship of C5orf34 expression with disease-specific survival in pan-cancer.** (**A**) The forest plots of C5orf34 in pan-cancer’s disease-specific survival; (**B**–**K**) C5orf34’s survival curves as regards disease-specific survival of pan-cancer samples.

**Figure 4:**
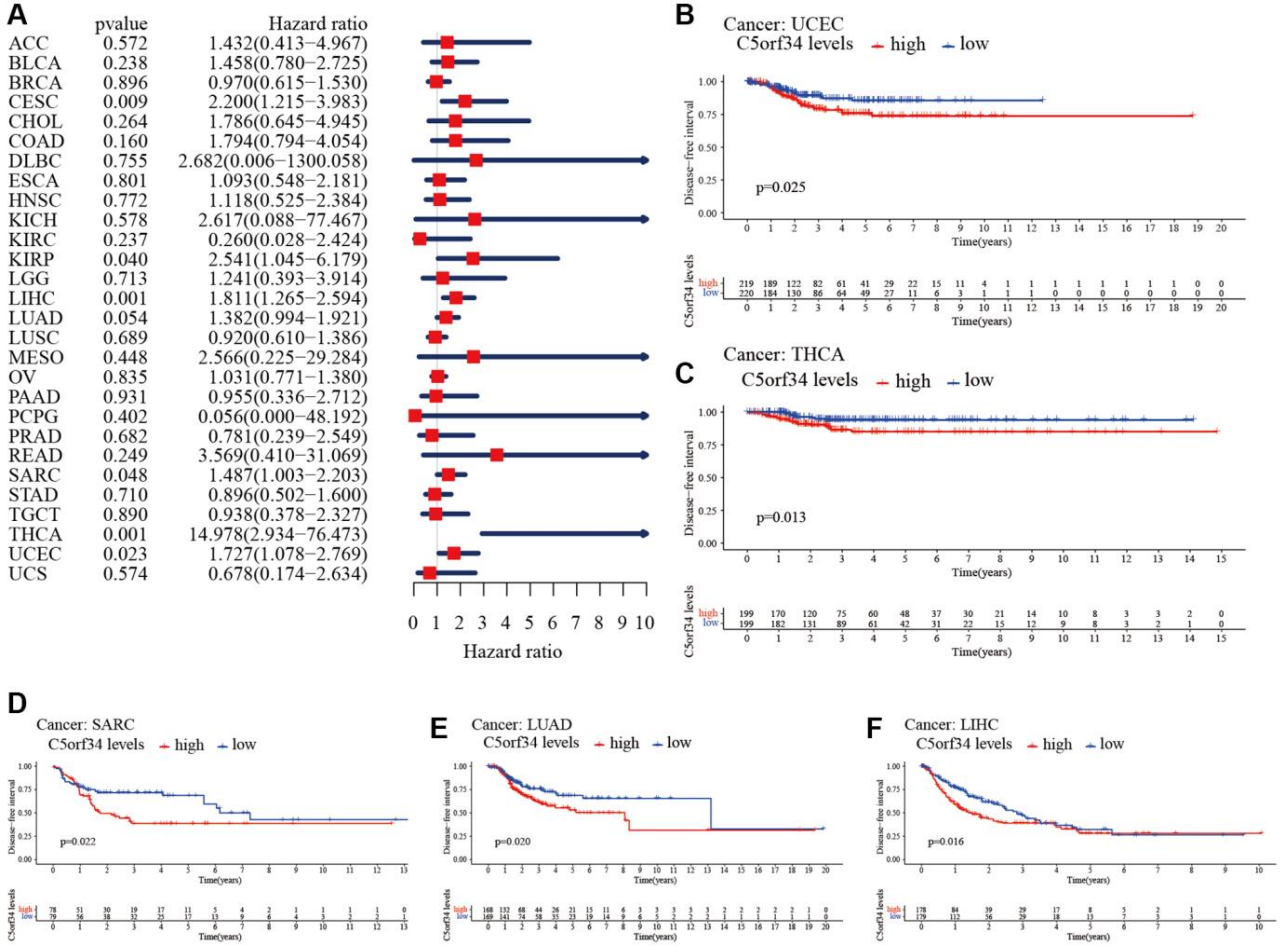
**Relationship of C5orf34 expression with disease-free interval in pan-cancer.** (**A**) The forest plots of C5orf34 in pan-cancer’s disease-free interval; (**B**–**F**) C5orf34’s survival curves as regards disease-free interval of pan-cancer samples.

**Figure 5:**
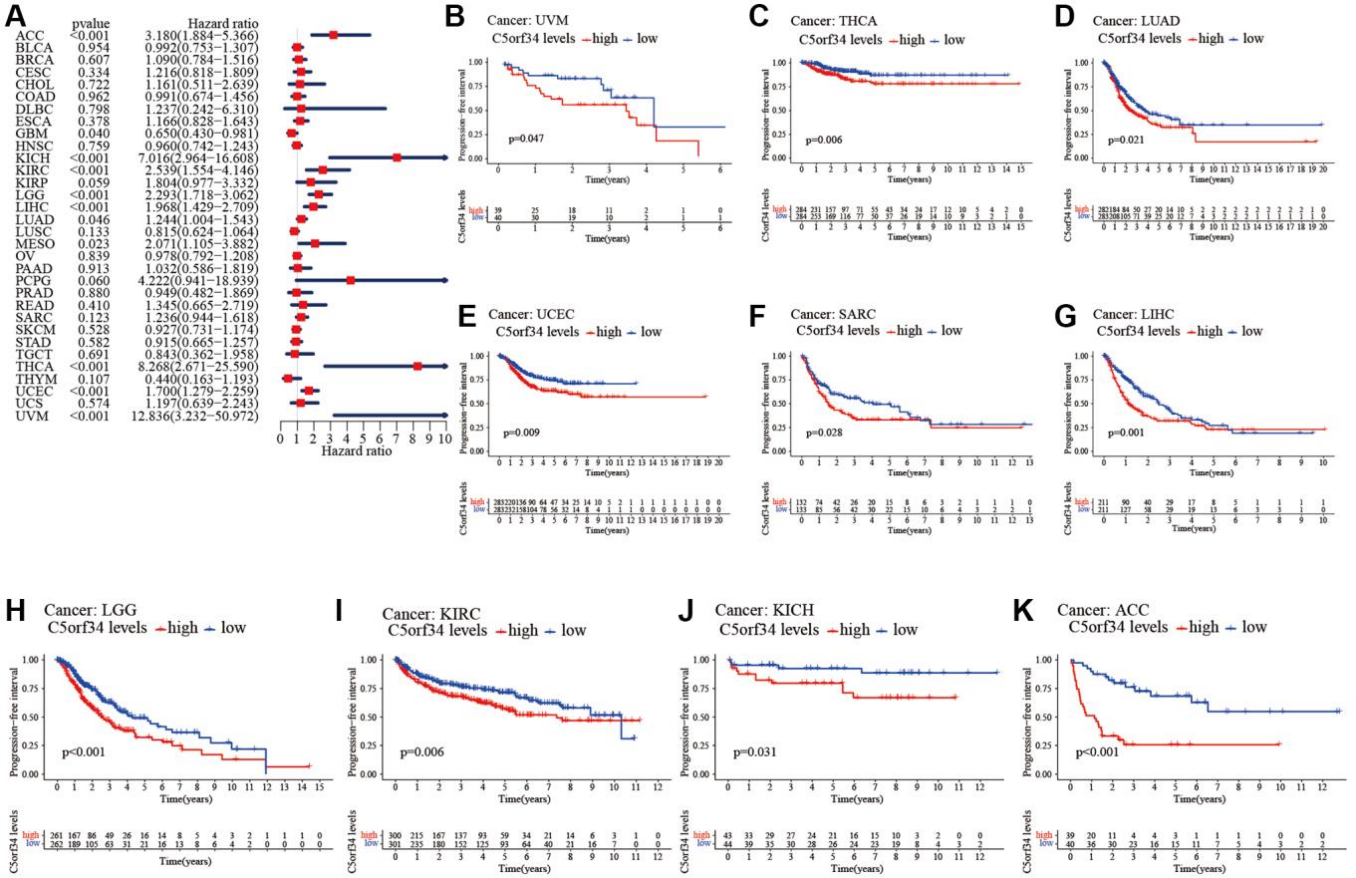
**Relationship of C5orf34 expression and progression-free interval in pan-cancer.** (**A**) The forest plots of C5orf34 in pan-cancer’s progression-free interval; (**B**–**K**) C5orf34’s survival curves as regards progression-free interval of pan-cancer samples.

With the aid of the data from TCGA database, the link between C5orf34 expression level and pan-cancer prognostic value was then explored. High mRNA expression was determined to be highly essential factor by the univariate Cox regression analysis of C5orf34-related survival in LGG, CESC, KIRP, LUAD, KIRC, THCA, LIHC, SARC, UCEC, ACC, KICH, THYM, PCPG, MESO and UVM (Figures 2A, 3A, 4A, 5A). Of note, high expression levels of C5orf34 led to a prolonged survival duration of patients with THYM (OS: *p* = 0.028), which was in line with the earlier findings from a Cox regression analysis (Figure 2A).

The Kaplan-Meier cumulative curves indicated elevated C5orf34 expression was significantly related to the worse OS, DSS and PFI of UVM, LIHC, UCEC, LGG, KIRC, KICH, and ACC (Figures 2B–2L; 3B–3K; 5B–5K). In addition, elevated C5orf34 expression was significantly related to the worse DFI of UCEC, THCA, SARC, LUAD, and LIHC (Figure 4B–4F).

### Relationship between clinical characteristics and C5orf34 expression in several cancers

Subsequently, we studied the correlation between mRNA expression and clinical features in multiple malignancies. LIHC, BRCA, THYM, HNSC, LUAD, CHOL, ESCA, and UCEC all exhibited substantial differences in C5orf34 expression in various age groups. In LIHC, BRCA, HNSC, LUAD, CHOL, ESCA, and THYM, decreased C5orf34 expression level was observed in patients more than 60 years old (Supplementary Figure 1). Furthermore, in the following six malignancies, the expression level of C5orf34 differed considerably by gender. In MESO, THCA, READ, KIRC, and HNSC, C5orf34 expression level was greater in the male group, but C5orf34 expression level was lower in the SARC male group (Supplementary Figure 1). Furthermore, C5orf34 expression differed by race in 5 cancers types including LIHC, UCEC, BRCA, ESCA, and ACC. Except for ACC, non-white patients had higher expression levels of C5orf34 (Supplementary Figure 1).

**Figure 6:**
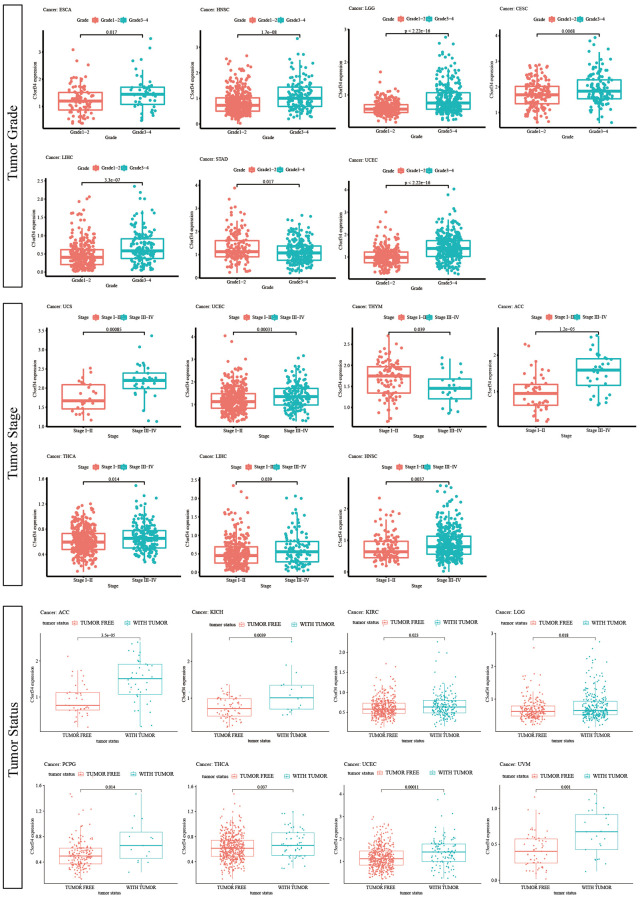
Relationship of C5orf34 expression with tumor stage, tumor grade, and tumor status.

In terms of the differences in C5orf34 expression by grade, it was discovered that C5orf34 was expressed more in G3-G4 patients in HNSC, ESCA, LIHC, LGG, UCEC, and CESC, and lower in patients of G3-G4 in STAD (Figure 6). In HNSC, UCS, LIHC, THCA, UCEC, and ACC, C5orf34 expression level appears to be greater in stage III-IV patients than in stage I and II patients, whereas C5orf34 expression level appears to be higher in stage I-II patients in THYM (Figure 6). Moreover, recurrent patients had a higher expression level of C5orf34 in KICH, LGG, PCPG, ACC, KIRC, UCEC, THCA, and UVM, as shown in Figure 6.

### Association between prognosis and C5orf34 methylation in several cancers

**Figure 7:**
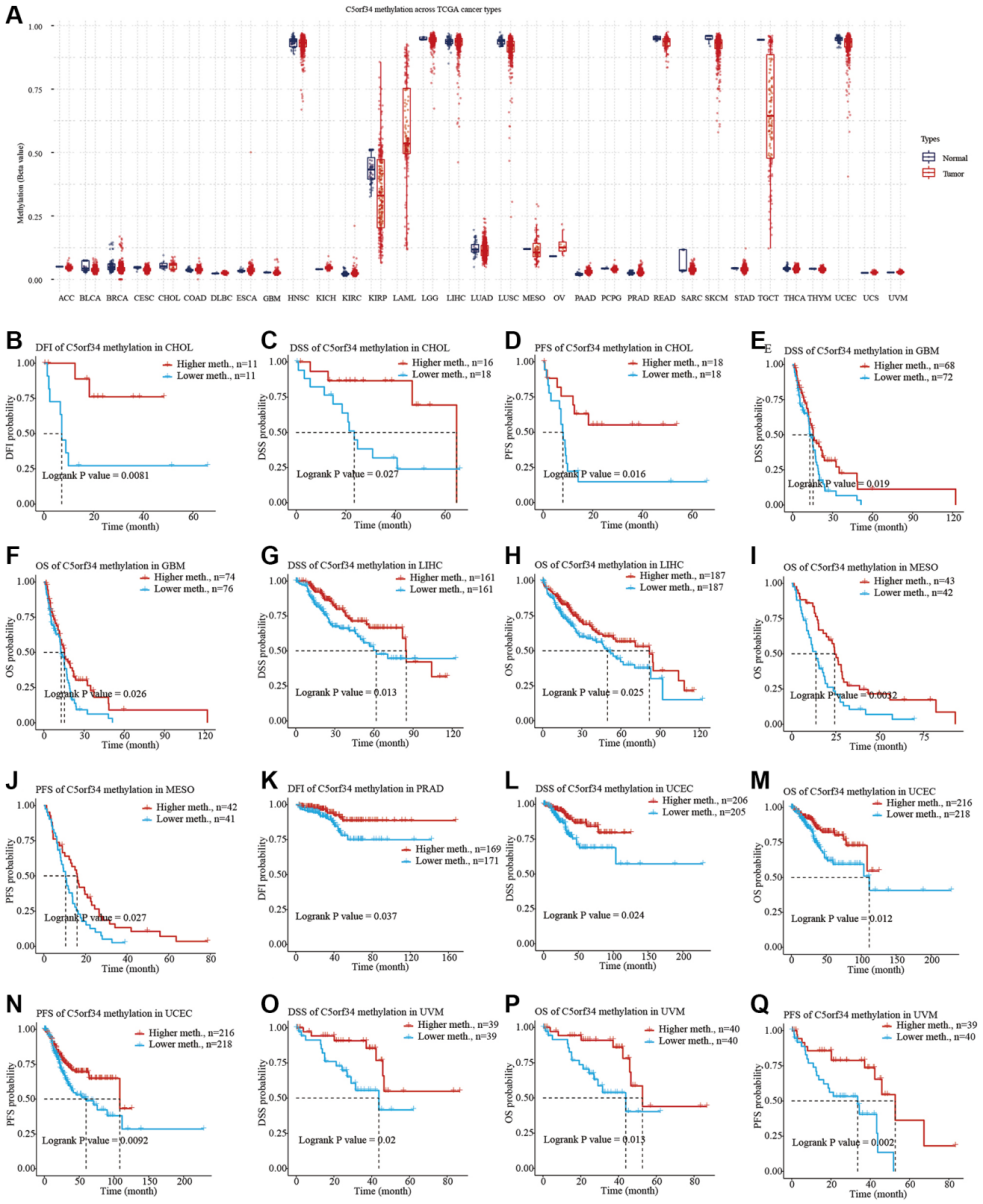
**C5orf34 methylation landscape on TCGA cancer types.** (**A**) The methylation level of C5orf34 differs dramatically between normal and malignant tissues; (**B**–**Q**) In UCEC, LIHC, PRAD, MESO, CHOL, GBM, and UVM, there was a correlation between C5orf34 methylation and patient prognosis.

We explored the potential relationship between C5orf34 methylation and numerous human malignancies after collecting data on C5orf34 methylation from the TCGA. With the exception of OV and PAAD, the majority of cancer tissues had lower C5orf34 methylation levels than normal tissues (Figure 7A). Furthermore, increased C5orf34 methylation levels were correlated with a favorable patient prognosis in several cancer patients. Patients with higher C5orf34 methylation levels had better DFI, PFS, and DSS in CHOL (Figure 7B–7D); on the other hand, patients with higher C5orf34 methylation levels had better DSS and OS in GBM (Figure 7E, 7F); patients with higher C5orf34 methylation levels had better DSS and OS in LIHC (Figure 7G, 7H); Patients with elevated C5orf34 methylation levels had prolonged PFS and OS duration in MESO (Figure 7I, 7J);, patients with elevated C5orf34 methylation levels had better DFI status in PRAD (Figure 7K);, patients with higher C5orf34 methylation had better PFS, DSS, and OS in UCEC (Figure 7L–7N); Patients with elevated C5orf34 methylation levels in UVM had prolonged OS, PFS, and DSS duration (Figure 7O–7Q).

### Copy number variation in C5orf34 in a variety of cancers

**Figure 8:**
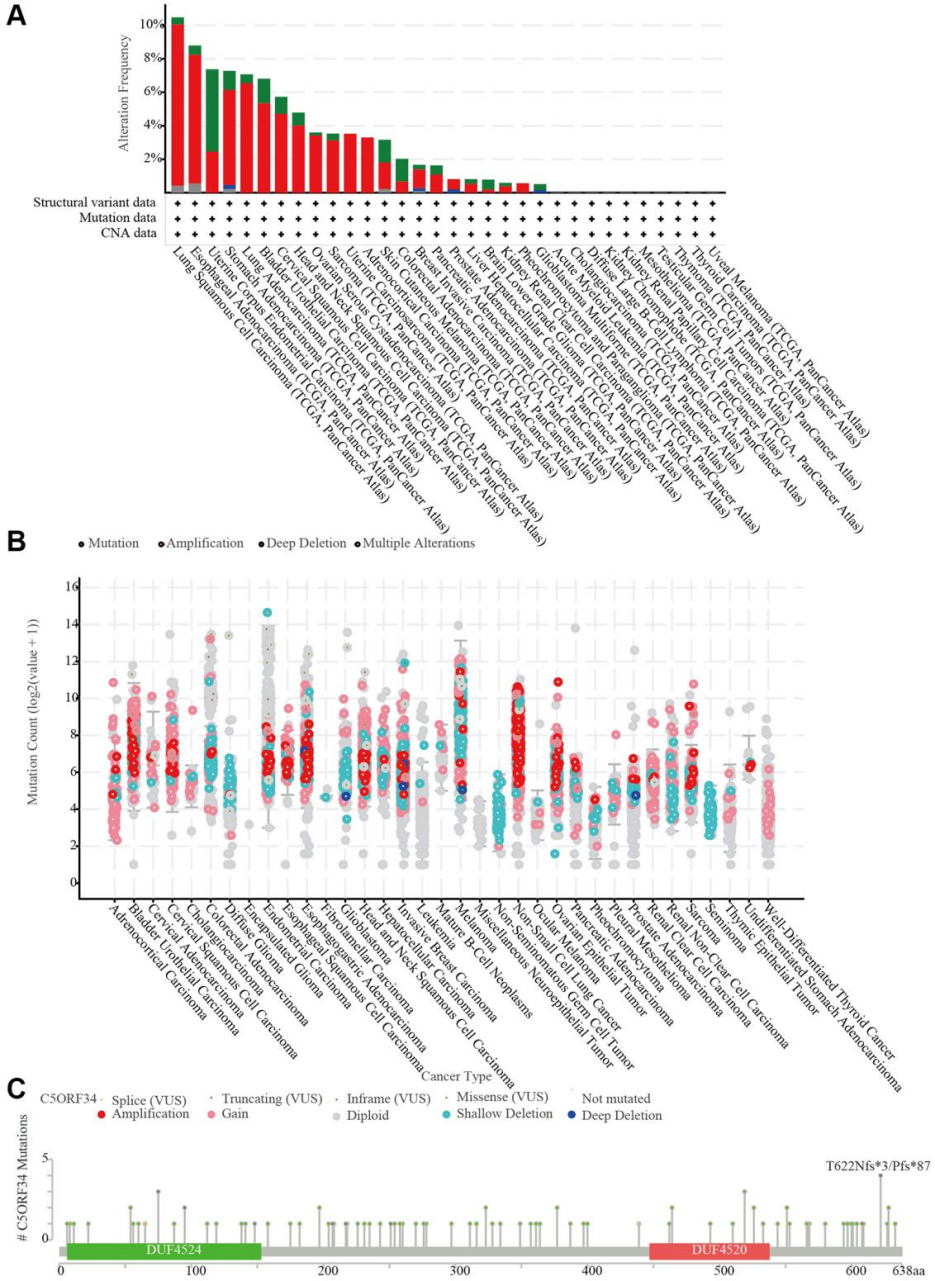
**C5orf34 mutations and copy number alterations.** (**A**) The cBioPortal database shows the mutation level of C5orf34; (**B**) The cBioPortal database shows the incidence of C5orf34 mutations in several TCGA pan-cancer investigations; (**C**) From the cBioPortal database, a mutation diagram of C5orf34 in several cancer types throughout protein domains.

CNV of critical genes has long been thought to play a role in the onset, progression, and patient prognosis of many malignancies. With the aid of the cBioPortal database, we explored the copy number variations of C5orf34. We discovered that the frequency of alteration was greater in esophageal adenocarcinoma and lung squamous cell carcinoma than in other malignancies. Amplification was the most common form of variation, followed by deep deletion and mutation (Figure 8A). The C5orf34 mutation count in a variety of malignancies was also presented in Figure 8B. Subsequently, the C5orf34 mutation types, locations, and case numbers were explored using the cBioPortal database. C5orf34 has 29 mutation locations (1 truncating and 28 missense) between amino acids 0 and 638 (Figure 8C).

### Relationship between C5orf34 variants and prognosis in several cancers

**Figure 9:**
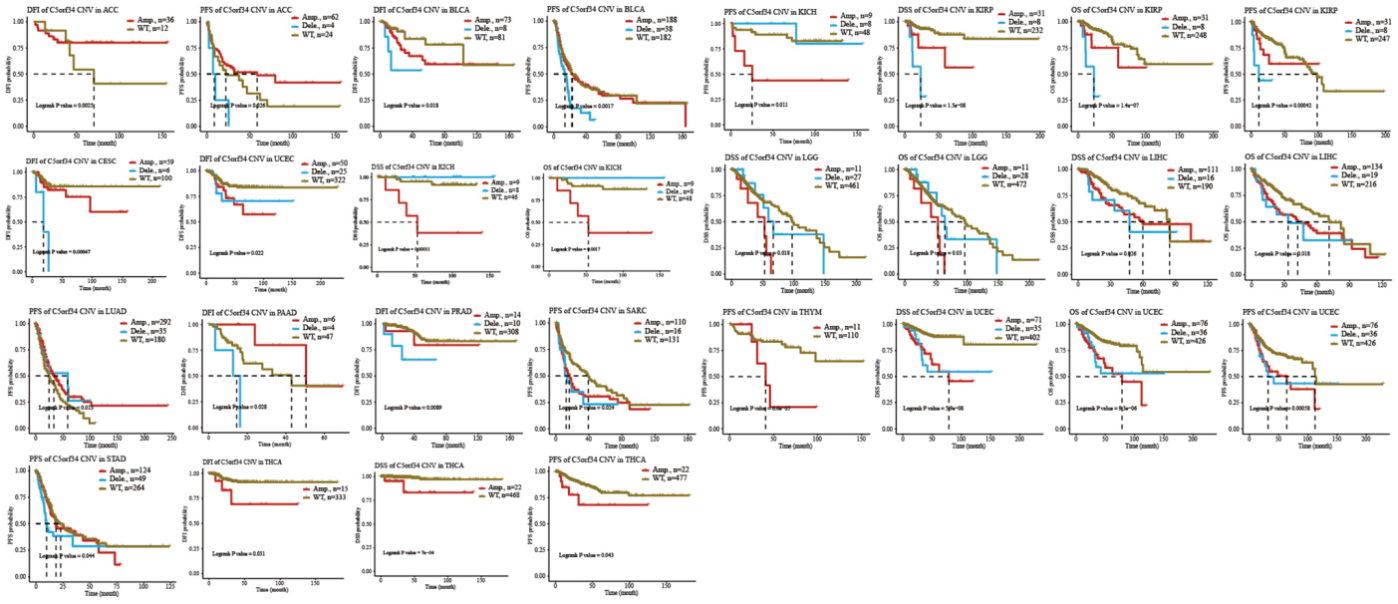
Relationship of C5orf34 copy number variation with prognosis in ACC, BLCA, CESC, KICH, KIRP, LGG, LIHC, LUAD, PAAD, PRAD, SARC, THYM, UCEC, STAD, and THCA.

The potential relationship between pan-cancer prognosis and C5orf34 variation was then further investigated. As depicted in Figure 9, ACC patients with amplification of C5orf34 exhibited a more favorable DFI status compared to those with the wild-type C5orf34. Additionally, they also demonstrated a superior PFS when contrasted with patients having C5orf34 deletion mutations or the wild-type variant. BLCA patients with deletion mutation had a shortened PFS and DFI duration in comparison to those with C5orf34 wide type or amplification. In comparison to patients with the wild-type C5orf34, individuals suffering from CESC and UCEC, who harbored C5orf34 deletion or amplification mutations, experienced a significantly reduced DFI duration. Patients with KICH who had C5orf34 deletion mutations exhibited an extended duration of PFS, OS, and DSS. In contrast, those who underwent C5orf34 amplification showed a poorer prognosis. In contrast to KIRP patients with C5orf34 amplification or the wild-type, those with C5orf34 deletion mutations experienced a significantly reduced duration of PFS, OS, and DSS. Patients with LGG who exhibited amplification of C5orf34 experienced a more unfavorable OS and DSS outcome. In LIHC patients, those with C5orf34 amplification, as well as those with deletion mutations, showed similar DSS and OS, both of which were worse when compared to individuals with the wild-type C5orf34. In the case of LUAD patients, those who had deletion mutations and amplification of C5orf34 experienced significantly improved PFS compared to individuals with the wild-type C5orf34. THCA patients with amplification of C5orf34 exhibited a more unfavorable prognosis, characterized by worse PFS, DSS, and DFI outcomes. UCEC patients with C5orf34 amplification or deletion mutations exhibited poorer survival outcomes compared to those with the wild-type C5orf34. For SARC, STAD, PAAD, PRAD, and THYM, patients who experienced amplification together with deletion mutation had worse prognosis in comparison to those with C5orf34 that is of wild type.

Furthermore, our study revealed the relevance of C5orf34 SNV to the clinical outcomes of several malignancies (Supplementary Figure 2). In the case of UCEC, mutant C5orf34 led to a longer OS and PFS duration compared to the wild-type C5orf34. Conversely, in CESC, mutant C5orf34 resulted in a poorer OS, DSS, and PFS status when compared to the wild-type C5orf34.

### Co-expression of C5orf34 with immune-related genes across various cancer types

**Figure 10:**
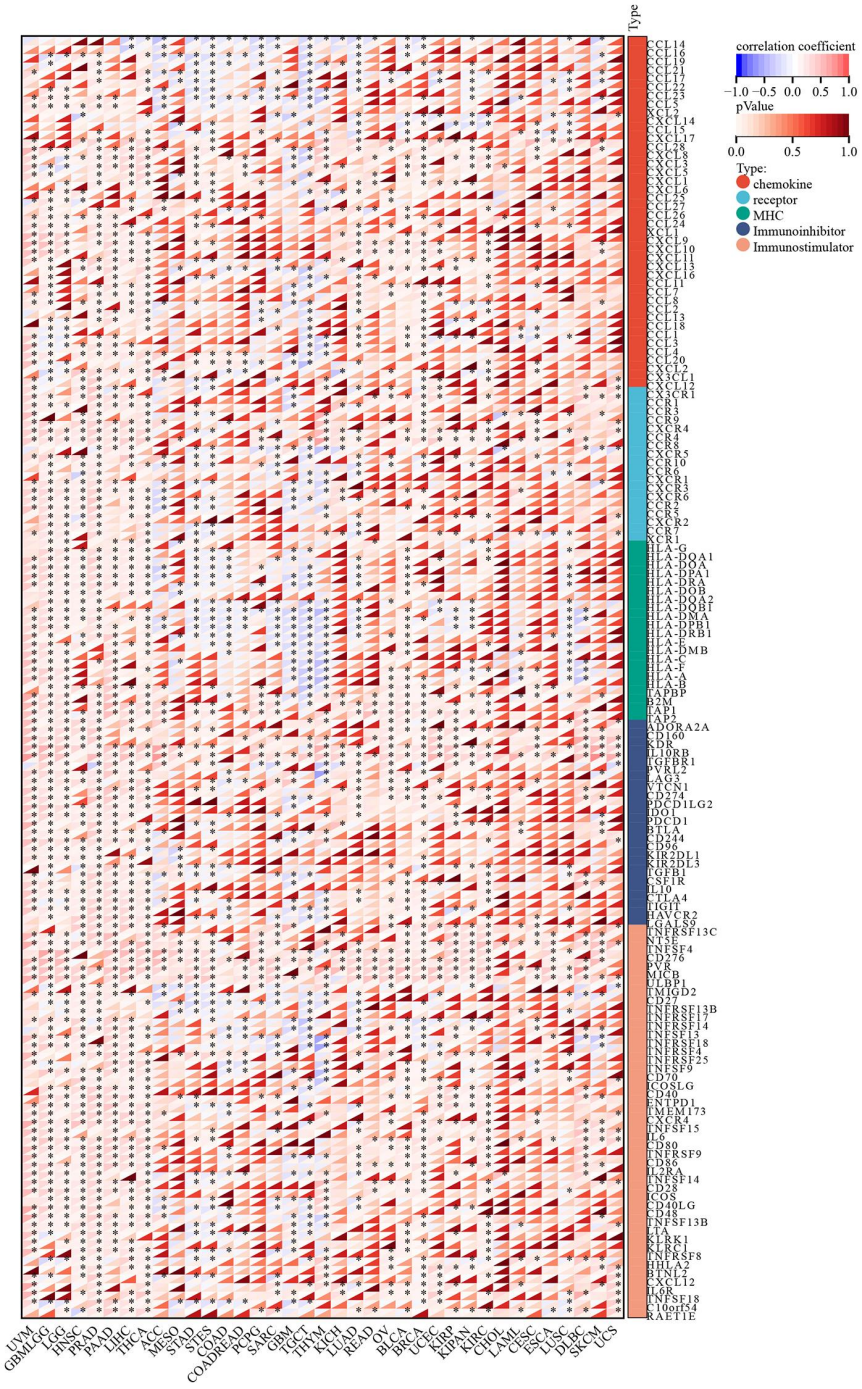
Relationship of C5orf34 expression with immune-related genes (including immunostimulators, chemokine receptors, chemokine ligands, immunoinhibitors, and genes encoding major histocompatibility complex).

The immune microenvironment within tumors is widely recognized for its complexity, and immune-related genes play a pivotal role as key regulators of this environment. In order to elucidate C5orf34’s immunomodulatory influence in the tumor microenvironment, we investigate the co-expression connection between immune-related genes and C5orf34 (including chemokine ligands, immune inhibitors, MHC, and chemokine receptors, and immunostimulators) in pan-cancer. Most immune-related genes were related to the expression of C5orf34 in the majority of malignancies, as illustrated in Figure 10. In the vast majority of malignancies, there was a positive correlation between the immune-related genes and C5orf34 expression. Notably, the expression level of C5orf34 exhibited a negative correlation with immune-related genes in THYM, TGCT, and GBM.

### Relationship between C5orf34 and immune cells infiltration in pan-cancers

**Figure 11:**
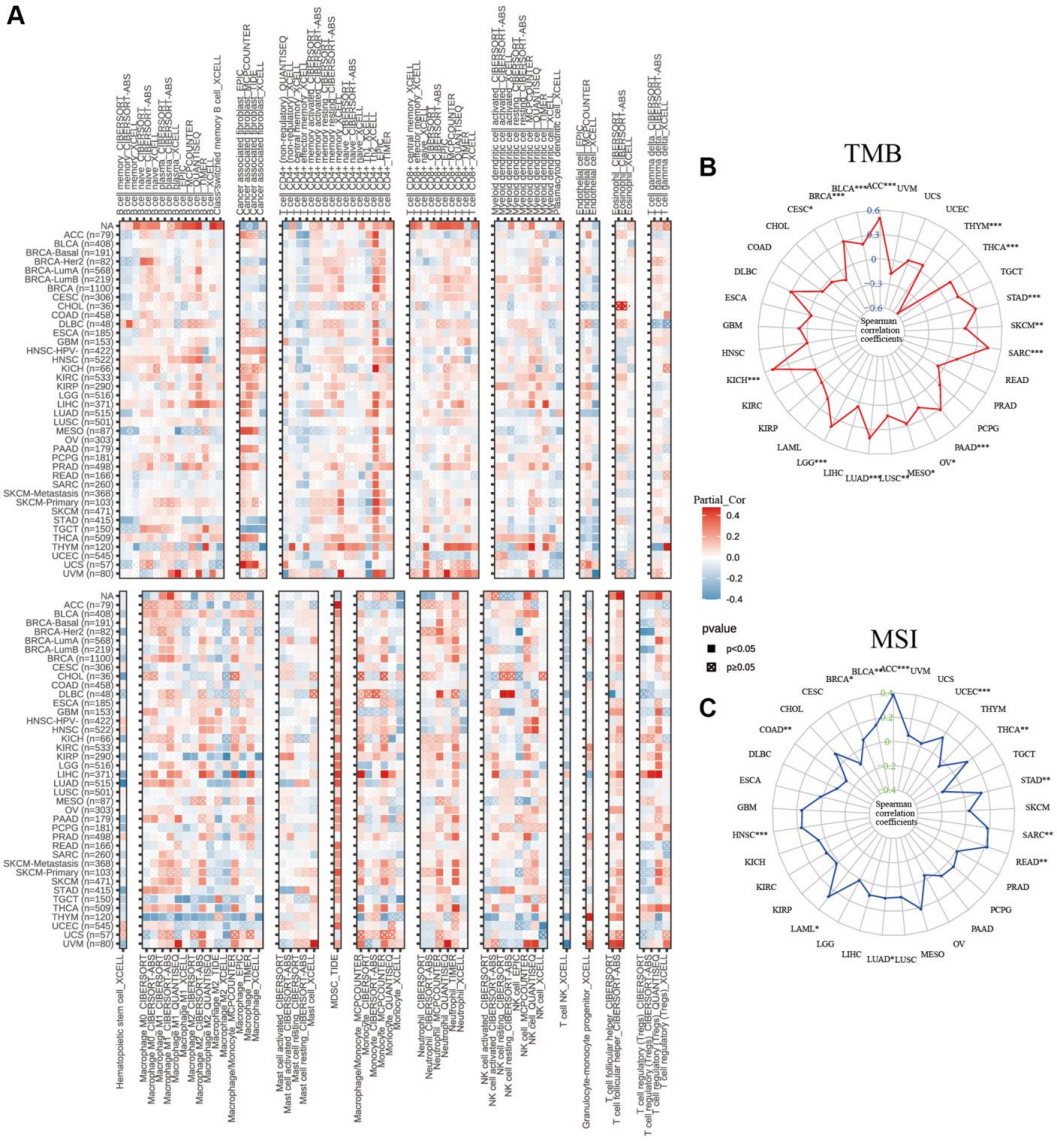
Relationship of C5orf34 expression wtih (**A**) immunocyte infiltration, (**B**) tumor mutation burden, and (**C**) microsatellite instability in pan-cancer.

In addition to the aforementioned immune-related genes, the infiltration of immune cells plays a pivotal role in the development and progression of malignant cancers. As a result, we investigated the interaction between infiltration of immune cells and expression of C5orf34 (Figure 11A). C5orf34 was shown to have a positive correlation with Th2 cells positively in a majority of malignancies, including SKCM, KICH, BLCA, ACC, and LIHC.

In THYM, C5orf34 exhibited a significant positive correlation with the majority of CD8+ T cells and CD4+ T cells, while showing a strong negative correlation with macrophages, including both M1 and M2 subtypes. In many cancers, including THYM, STAD, UVM, and OV, T cell follicular helper cells displayed a positive correlation with C5orf34, except in the case of DLBC, where a negative correlation was observed. In THYM and LIHC, there was a favorable correlation between C5orf34 and T cell regulatory (Tregs). In BRCA, regarding the correlation with Tregs, a positive association was noted in BRCA-LumA, while a negative correlation was observed in BRCA-LumB and BRCA-Her2.

The expression of C5orf34 was positively correlated with TMB in SARC, ACC, STAD, KICH, PAAD, BRCA, THCA, LUAD, LGG, and BLCA. Nonetheless, an inverse association with TMB was observed in the THYM cohorts (Figure 11B). Among the READ, THCA, STAD, SARC, ACC, HNSC, UCEC, and BLCA cohorts, tumors with a high Microsatellite Instability (MSI-High) status exhibited higher C5orf34 expression levels compared to those with genetic stability. However, the inverse trend was only observed in the COAD cohorts (Figure 11C). Despite the strength of these associations, the correlation coefficients between TMB and C5orf34, together with MSI, were below 0.6 in nearly all malignancies, suggesting that C5orf34 was not expected to affect tumorigenesis by playing a role in the genetic alterations process and that it was not adequate to predict the patient’s response to immune-checkpoint blockade (ICB) either by its own.

### Tumor microenvironment and stemness indices in pan-cancers

**Figure 12:**
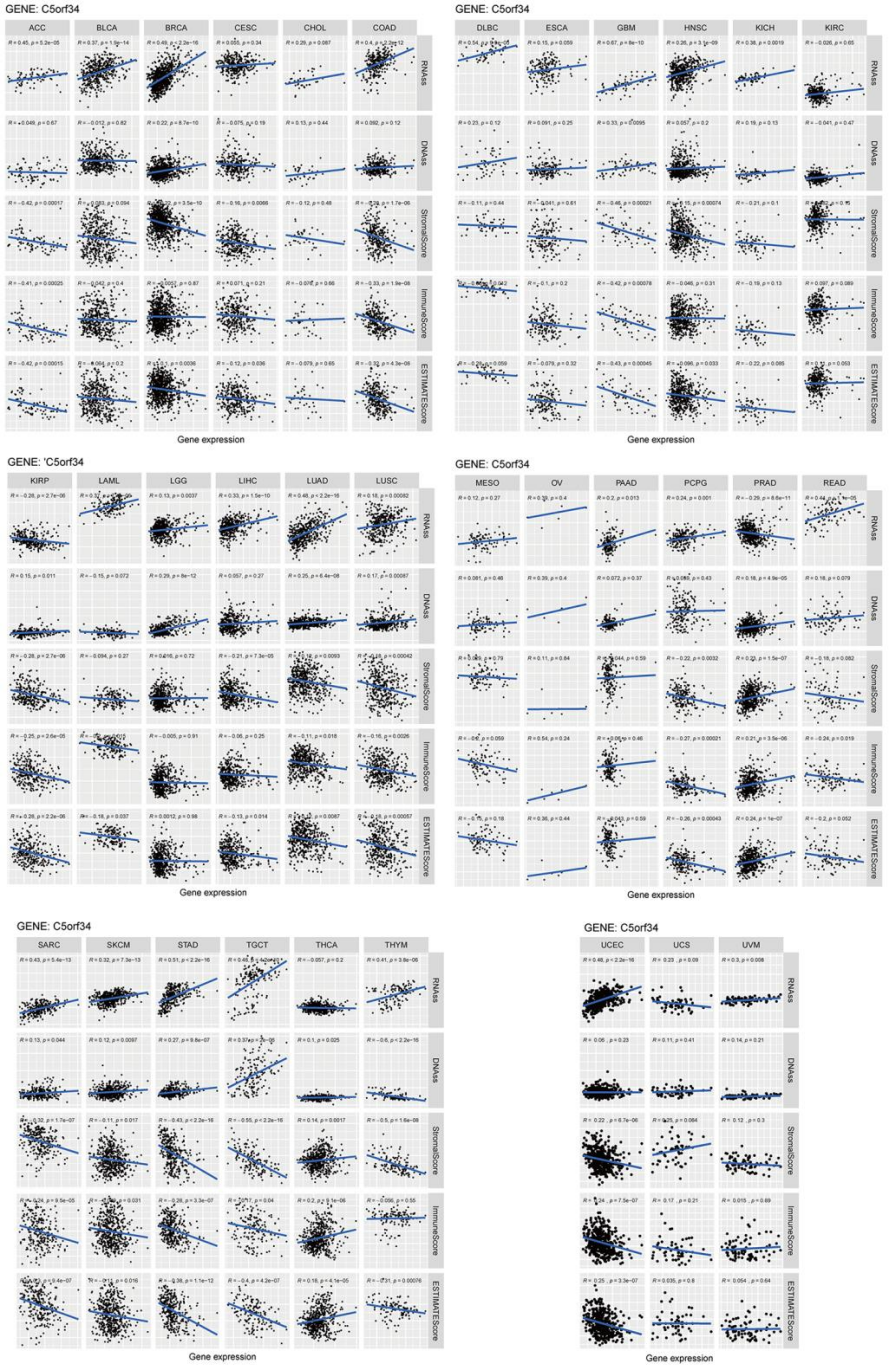
Relationship of C5orf34 expression with stromal scores, RNA stemness scores, DNA stemness scores, immune scores, and ESTIMATE scores in pan-cancer.

To thoroughly comprehend the relationship between C5orf34 and pan-cancers stemness, researchers employed DNAss and RNAss. C5orf34 exhibited a high positive correlation with both RNAss and DNAss in GBM, BRAC, LGG, SKCM, LUSC, STAD, LUAD, and TGCT (all *p* < 0.01). Notably, C5orf34 was found to have a negative correlation with RNAss in PRAD and KIRP. Additionally, it displayed a negative correlation with DNAss in THYM. (Figure 12). The ESTIMATE technique was used to calculate ESTIMATEScore, ImmuneScore, and StromalScore for the purpose of performing further investigations of the tumor microenvironment. Figure 12 shows the relationship between the ratio of stromal and immune cells and C5orf34 expression in TCGA tumor samples. C5orf34 exhibited an adverse association with the estimated score, immune score, and stromal score in STAD, PCPG, TCGT, SARC, SKCM, LUAD, LUSC, COAD, GBM, KIRP, ACC, and UCEC. C5orf34 showed a favorable relationship with the estimated score, immune score, and stromal score in PRAD and THCA.

### Single-cell analysis of C5orf34 in female reproductive system tumors

With the help of miRNet, miRTarBase, and StarBase databases, a total of 47 miRNAs were found to be closely related to C5orf34, which established the framework for future study (Supplementary Tables 2–4). The single-cell results of GSE118828 showed that OV micro-environment was composed of conventional CD4+ T cells, endothelial, fibroblasts, malignant cells, monocyte/macrophage, and myofibroblasts (Supplementary Figure 3A). The expression of C5orf34 is predominantly observed in certain immune cells of OV, such as conventional CD4 T cells and monocyte/macrophage (Supplementary Figure 3A). The single-cell results of UCEC based on GSE139555 revealed that UCEC micro-environment was composed of conventional CD4+ T cells, CD8+ T cells, exhausted CD8+ T cells, fibroblasts, Tprolif, and Treg cells (Supplementary Figure 3B). C5orf34 expression is relatively uniform across various types of immune cells in UCEC (Supplementary Figure 3B). The single-cell results of GSE168652 showed that CESC micro-environment was composed of CD8+ T cells, stromal cells, endothelial, fibroblasts, malignant cells, monocyte/macrophage, and smooth muscle cell (Supplementary Figure 3C). Similar to UCEC, various cell types in CESC exhibit similar C5orf34 expression patterns (Supplementary Figure 3C).

## DISCUSSION

With the aid of the independent GTEx, TCGA, and Oncomine databases, we examined the C5orf34 expression in 33 distinct cancer types in the present research, demonstrating significant disparities in a pan-cancer expression of C5orf34 between normal and tumor tissues. In addition, we discovered a correlation between C5orf34 expression and immune cell infiltration, prognosis, clinical features, tumor microenvironment, and immunotherapy indicators (TMB, MSI) in the present research. Eventually, we discovered that C5orf34 expression has a close relationship with the prognosis of patients with cancer, which may open up new avenues for clinical research.

To begin, we used the independent GTEx, TCGA, and Oncomine databases for the purpose of acquiring data on the C5orf34 expression in 33 distinct cancers. Most tumors include kidney cancer, liver cancer, brain, and central nervous system cancer, gastric cancer, bladder cancer, leukemia, colorectal cancer, ovarian cancer, head and neck cancer, lymphoma, esophageal cancer, breast cancer, sarcoma, pancreatic cancer, cervical cancer, lung cancer, prostate cancer, melanoma, and other cancer, had elevated C5orf34 levels than normal tissues, according to Oncomine data. However, in several other datasets, there were lowered C5orf34 levels in some cancers, including breast cancer, leukemia, lymphoma, ovarian cancer, brain, and central nervous system cancer, prostate cancer, and other cancers. The expression level of C5orf34 was highly elevated in LUSC, KIRC, STAD, CHOL, COAD, KIRP, BLCA, LIHC, HNSC, READ, LUAD, BRCA, GBM, ESCA, and UCEC, but lowered in PRAD, and in the meantime, there was no difference that was significant in THCA in comparison to the normal tissues, according to analysis results obtained based on TCGA and GTEx data. Moreover, in pan-cancer, we intensively explored C5orf34’s predictive relevance. In this study, we discovered that increased mRNA expression of C5orf34 was associated with an unfavorable prognosis in patients with CESC, KIRP, SARC, LIHC, MESO, THCA, THYM, KIRC, ACC, GBM, PCPG, KICH, LGG, UCEC, LUAD, and UVM. Patients with THYM who exhibited high C5orf34 expression levels were observed to have a longer survival time. In LUAD, C5orf34 could potentially contribute to the development and advancement by modulating the MAPK signaling pathway [4]. C5orf34 has a malignant biological characteristic and complex prognostic value for pan-cancer, according to these findings.

Second, we investigated the correlation between clinical features and C5orf34 expression. C5orf34 expression is substantially related to race, gender, age, and pathologic-grade, according to our findings. In HNSC, LGG, UCEC, LIHC, ESCA, and CESC, we discovered that in G3-G4 grade patients, the expression level of C5orf34 was higher, but lower in G3-G4 patients in STAD. In THCA, UCS, UCEC, LIHC, HNSC, and ACC, C5orf34 expression level is greater for patients in stage III-IV than for those in stage I and II. However, in THYM, the expression level of C5orf34 has been elevated in stage I-II patients. These findings suggested that C5orf34 may have a role in the onset and progression of cancer.

Following that, we delved into C5orf34 methylation in pan-cancer. The most well-studied epigenetic change in humans, DNA methylation, was also the first to be discovered in cancer [26, 27]. DNA methylation plays a crucial role in a variety of biological activities, and it has been related to cancer. Normal and malignant cells do, as a matter of fact, have distinct methylomes. In most cancers, a worldwide hypomethylation pattern is found, resulting in instability of oncogenes activation [28]. Except for PAAD and OV, we discovered that most cancer tissues contained lower C5orf34 methylation levels in comparison to the normal tissues (Figure 12). Furthermore, several cancer patients who had elevated C5orf34 methylation levels had better prognoses, including MESO, UCEC, LIHC, CHOL, UVM, GBM, and PRAD. Many other reports also revealed that the DNA methylation was deregulated in pan-cancer and was correlated with unfavorable prognoses [29].

Human malignancies are characterized by chromosome CNVs, which include genes that are in hundreds or thousands [3, 17]. According to a growing amount of evidence, CNVs are recurring in several cancer types, with some of them having a probability of appearing in the early tumorigenesis stages, demonstrating that some cancer-associated CNVs may serve as human cancers drivers [30, 31]. CNAs can speed up tumor growth by changing the gene expression levels whose location is at regions of the impacted genomic [32]. CNVs are also a major source of genetic disorders where they account for 5–9% of the pathogenic/likely pathogenic variations in multigene panel testing for a variety of disorders [33, 34]. Many studies have revealed a relationship between certain CNVs to tumor development, invasion, and prognosis, including NSCLC [35], serous ovarian cancer [36], and breast cancer [37]. We discovered that amplification was found to be the most common form of variation in the present research. In addition to this, compared to other cancers, the frequency of alteration was higher in esophageal adenocarcinoma and lung squamous cell carcinoma in comparison to other cancers. Notably, we discovered that C5orf34 SNV was closely related to patient prognosis in a variety of cancers.

Tumor Microenvironment (TME) is a complicated ecosystem made up of stromal, malignant, and immune cells [38]. The TME cellular components frequently coevolve with the tumor. Furthermore, alterations in TME may shift the immune milieu’s balance favoring immunosuppression, granting permission to tumor evasion together with evasion of immune surveillance. Immune-related genes are also crucial regulators of the tumor microenvironment. As a result, we investigated the co-expression relationship between immune-related genes and C5orf34. We discovered that the majority of the immune-related genes had a relationship with the expression of C5orf34 and that a positive relationship was observed between the immune-related genes and C5orf34 expression (including immunoinhibitors, chemokine receptors, MHC, chemokine ligands, and immunostimulators) in most of the several cancers (Figure 10). Immune cells that infiltrate tumors play a crucial role in tumor growth as well as the response of immunotherapeutic [39]. The composition of tumor-infiltrating cells indicates the processes behind anticancer immune responses, and they can also aid in the discovery of new prognostic markers. Another important conclusion of this research is that the expression of C5orf34 is strongly related to immune infiltration. In various cancers, the expression of C5orf34 has a positive correlation with the high presence of immune infiltrates, particularly the T cell follicular helper, Th2 cells, and Tregs. Moreover, we discovered that C5orf34 was associated with Th2 cells in a positive manner in several cancers, including ACC, KICH, BLCA, SKCM, and LIHC. In most cancer such as STAD, THYM, UVM, and OV, C5orf34 was shown to have a positive correlation with T cell follicular helper, as well as a positive correlation with Tregs in LIHC and THYM. C5orf34, on the other hand, demonstrated a favorable relationship with most CD8+ T and CD4+T cells in THYM but a substantial negative correlation with macrophages, which include M1 and M2. In BRCA, between C5orf34 and Tregs, a positive correlation was observed in BRCA-LumA, but a negative correlation was observed in BRCA-Her2 and BRCA-LumB.

Tumor mutation burden (TMB) is a self-determining biomarker that has recently been found in a number of tumor immunotherapies and may be used in predicting immunotherapy efficacy [40, 41]. Therapy of immune checkpoint inhibitor has been demonstrated to assist the treatment of patients with high TMB [42] MSI is correlated with the incidence, progression, and patient prognosis of numerous malignancies and plays a crucial role in malignant tumors pathogenesis. Patients with high MSI levels have a superior anti-tumor immune response, the capacity to limit tumor cell proliferation, and a better prognosis than those with low MSI levels [42–45]. As a result, MSI has the capability of serving as a crucial predictor in assessing the malignancy degree, tumors prognosis, and efficacy [46]. The findings of this study regarding the relationship between TMB, C5orf34, and MSI demonstrate that TMB and MSI and C5orf34 expression are positively correlated in a variety of cancer types, including THCA, LUAD, LGG, KIHC, BRCA, SARC, PAAD, STAD, ACC, and BLCA. In nearly all cancer types, the correlation coefficients between TMB, MSI, and C5orf34 were consistently below 0.6. This suggests that C5orf34 is unlikely to significantly influence tumorigenesis through genetic alterations and may not be a strong predictor of patient response to immune checkpoint blockade (ICB).

Immunotherapy is a powerful treatment option for patients with treatment-resistant cancer, and the TME components have a remarkable impact on the therapeutic response [47, 48]. By computing the immune/stromal/Assess score, the ESTIMATE method is an extensively acknowledged and robust method used in several malignancies in estimating the immune cells/infiltrating stromal level and the purity of tumor. TME has been characterized with the aid of the ESTIMATE algorithm in a variety of solid cancers, such as osteosarcoma [49], cervical squamous cell carcinoma [50], endometrial cancer [51], and breast cancer [52]. It was extensively shown to predict infiltration of nontumor cells successfully in TME. The ESTIMATE method was utilized in investigating the correlation between the expression of C5orf34 and the stromal and immune cells ratio. In ACC, SARC, PCPG, COAD, LUSC, KIRP, GBM, SKCM, STAD, LUAD, TCGT, and UCEC, C5orf34 was found to contain a negative correlation with the immune score, stromal score, and estimated score; however, the correlation was positive with the immune score, stromal score, and estimated score in THCA and PRAD.

Despite the fact that this study verified the role of C5orf34 in tumor formation and patient prognosis, certain levels of limitations remained. The information is derived solely from public databases and has not been tested experimentally in any way. C5orf34 is also strongly expressed in several cancers and is related to an unfavorable prognosis, although the precise molecular mechanism behind this effect has not been determined. There is a correlation between C5orf34 expression and TMB, tumor immunity, tumor microenvironment, and MSI; however, there isn’t enough evidence to prove it. As a result, more research into the functional processes of C5orf34 in malignancies is warranted.

In spite of this first-ever synopsis, which highlights C5orf34’s crucial involvement in pan-cancer, it is vital to note that there are still certain limitations to be aware of. Due to the limited sample size, more large-scale prospective studies are required to confirm the conclusions of our research. In addition, we only conducted experimental verification at the level of gene expression; in the future, more in-depth fundamental investigations will be required to examine and confirm the precise mechanism of C5orf34 on carcinogenesis and development.

## CONCLUSIONS

The expression level of C5orf34 was found to be elevated in several cancers, and this high expression is highly related to poor survival in most tumors. The immunotherapy indicators, immune cell infiltration, and tumor microenvironment (TMB, MSI) have a correlation with the expression level of C5orf34. Finally, our data corroborate the role of C5orf34 expression in tumor prognosis and provide fresh information about cancer treatment choices.

## Supplementary Materials

Supplementary Figures

Supplementary Tables
