# Supplementary material for: Integration of genomics and transcriptomics highlights the crucial role of chromosome 5 open reading frame 34 in various human malignancies
Source: Aging (Albany NY). 2023 Dec 7;15(23):14384–410. doi: 10.18632/aging.205310 (PMC10756085; doi:10.18632/aging.205310)
Supplement: Supplementary Figures [file aging-15-205310-s001.pdf]

SUPPLEMENTARY FIGURES

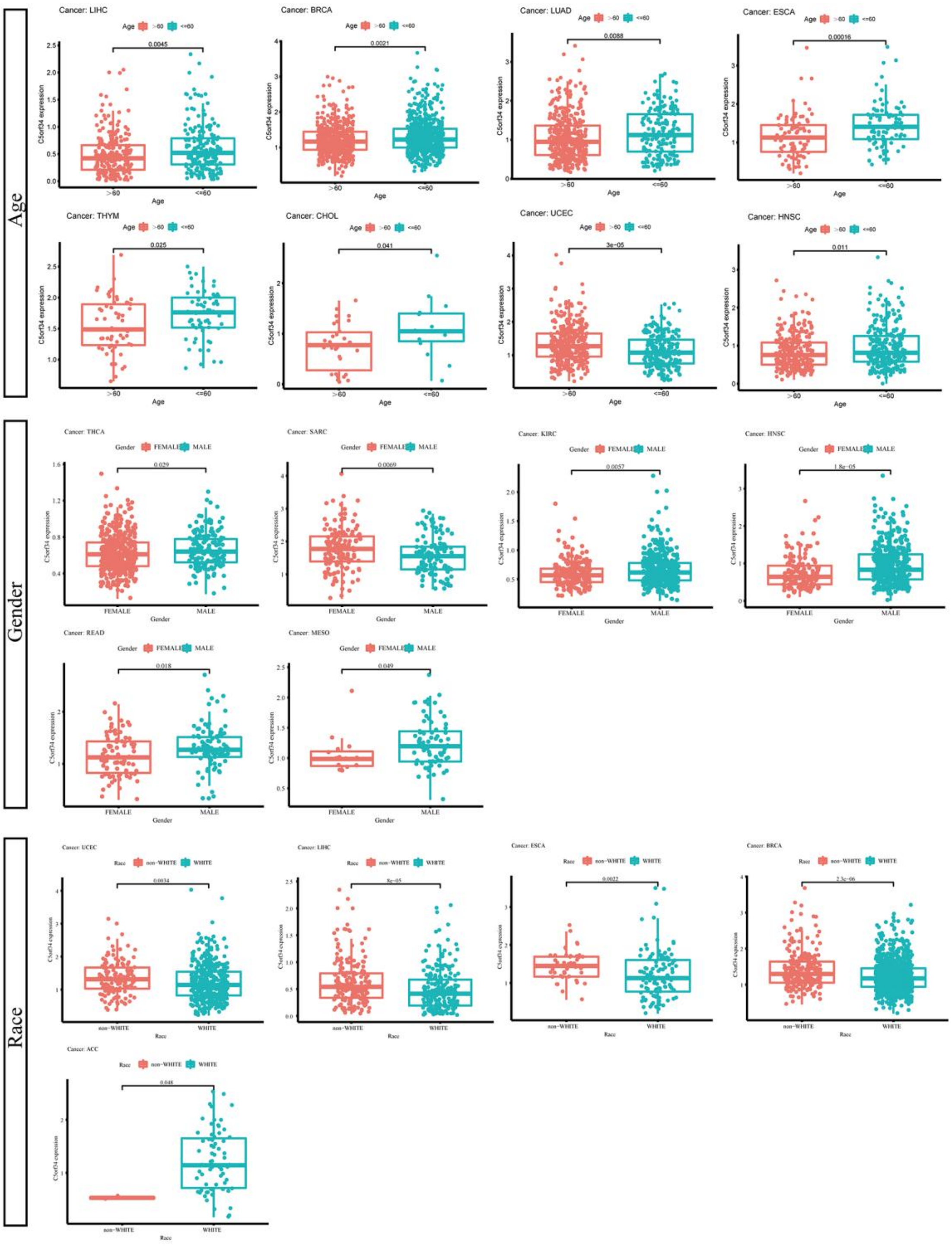

Supplementary Figure 1. Relationship of C5orf34 expression with gender, age, and race in cancers.

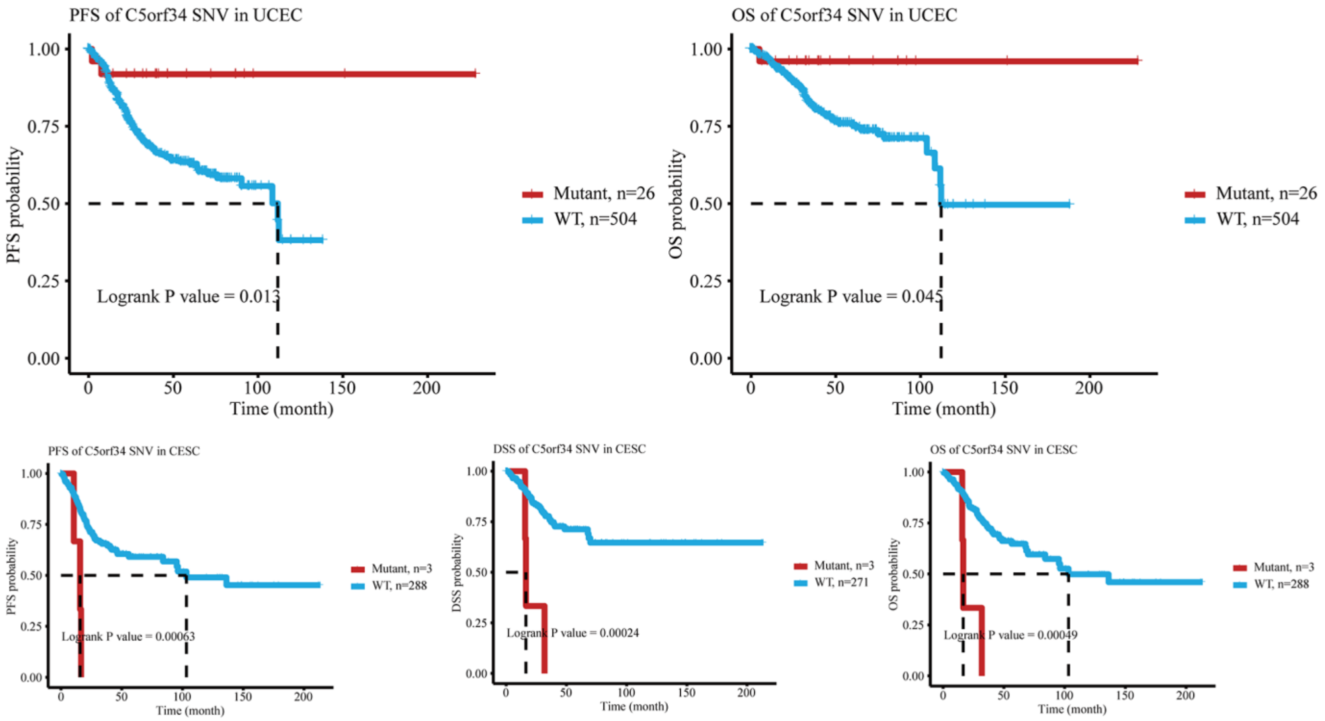

Supplementary Figure 2. Relationship of C5orf34 single-nucleotide variant with prognosis in UCEC and CESC.

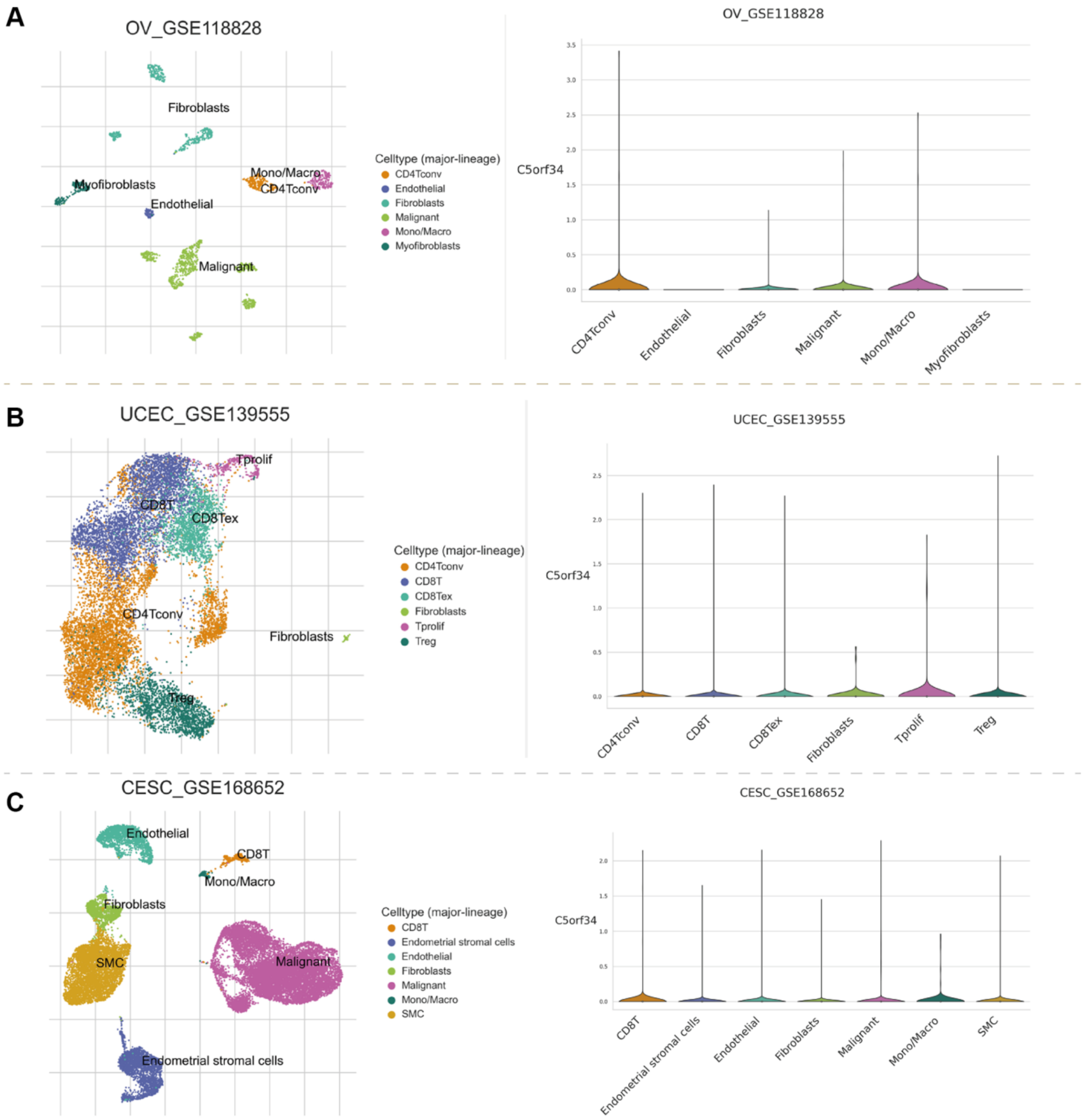

Supplementary Figure 3. Single-cell analysis of C5orf34 in (**A**) OV, (**B**) UCEC, and (**C**) CESC based on TISCH2 platform.
