# Supplementary material for: Integration of genomics and transcriptomics highlights the crucial role of chromosome 5 open reading frame 34 in various human malignancies
Source: Aging (Albany NY). 2023 Dec 7;15(23):14384–410. doi: 10.18632/aging.205310 (PMC10756085; doi:10.18632/aging.205310)
Supplement: Supplementary Tables [file aging-15-205310-s002.pdf]

SUPPLEMENTARY TABLES

Supplementary Table 1. The abbreviations of cancer types.

Cancer type	Abbreviations
Adrenocortical carcinoma	ACC
Bladder Urothelial Carcinoma	BLCA
Breast invasive carcinoma	BRCA
Cervical squamous cell carcinoma	CESC
Cholangiocarcinoma	CHOL
Colon adenocarcinoma	COAD
Lymphoid Neoplasm Diffuse Large B-cell Lymphoma	DLBC
Esophageal carcinoma	ESCA
Glioblastoma multiforme	GBM
Head and Neck squamous cell carcinoma	HNSC
Kidney Chromophobe	KICH
Kidney renal clear cell carcinoma	KIRC
Kidney renal papillary cell carcinoma	KIRP
Acute Myeloid Leukemia	LAML
Brain Lower Grade Glioma	LGG
Liver hepatocellular carcinoma	LIHC
Lung adenocarcinoma	LUAD
Lung squamous cell carcinoma	LUSC
Mesothelioma	MESO
Ovarian serous cystadenocarcinoma	OV
Pancreatic adenocarcinoma	PAAD
Pheochromocytoma and Paraganglioma	PCPG
Prostate adenocarcinoma	PRAD
Rectum adenocarcinoma	READ
Sarcoma	SARC
Skin Cutaneous Melanoma	SKCM
Stomach adenocarcinoma	STAD
Testicular Germ Cell Tumors	TGCT
Thyroid carcinoma	THCA
Thymoma	THYM
Uterine Corpus Endometrial Carcinoma	UCEC
Uterine Carcinosarcoma	UCS
Uveal Melanoma	UVM

Supplementary Table 2. The prediction results from the miRNet database.

hsa-mir-192-5p
hsa-mir-181b-5p
hsa-mir-125b-2-3p
hsa-mir-148b-5p
hsa-mir-22-5p
hsa-mir-27a-3p
hsa-mir-27b-3p
hsa-mir-29b-2-5p
hsa-mir-30a-3p

hsa-mir-30d-3p
hsa-mir-30e-3p
hsa-mir-412-3p
hsa-mir-455-5p
hsa-mir-4705
hsa-mir-518a-3p
hsa-mir-590-3p
hsa-mir-1-3p
hsa-mir-1343-3p
hsa-mir-103a-3p
hsa-mir-107
hsa-mir-124-3p
hsa-mir-128-3p
hsa-mir-129-2-3p
hsa-mir-147a
hsa-mir-195-5p
hsa-mir-210-3p
hsa-mir-26a-5p
hsa-mir-34a-5p
hsa-mir-376a-5p
hsa-mir-483-5p

Supplementary Table 3. The prediction results from the miRTarBase database.

hsa-miR-192-5p
hsa-miR-181b-5p

Supplementary Table 4. The prediction results from the StarBase database.

hsa-miR-205-5p
hsa-miR-223-3p
hsa-miR-362-5p
hsa-miR-369-3p
hsa-miR-325
hsa-miR-410-3p
hsa-miR-494-3p
hsa-miR-520g-3p
hsa-miR-520h
hsa-miR-655-3p
hsa-miR-668-3p
hsa-miR-340-5p
hsa-miR-889-3p
hsa-miR-875-5p
hsa-miR-513b-5p
hsa-miR-1323
hsa-miR-1323
hsa-miR-548o-3p
